# A Multi-layer Linguistic Profiling Framework for Emotion Recognition in Text: A Data-Driven Natural Language Processing (NLP) Study Using the Kaggle Emotions Dataset

**DOI:** 10.7759/cureus.115651

**Published:** 2026-09-02

**Authors:** Roaa S Bogdadi, Marivel M De Guzman, Rahaf Al Hasheem, Mohammad Bani Abed Alghani

**Affiliations:** 1 Research, King Abdullah Medical Complex, Jeddah, SAU; 2 Research, Research Assistant (RA) Company for Research and Studies, Riyadh, SAU; 3 Data Science/AI, Pioneers Academy, Amman, JOR

**Keywords:** emotion recognition, explainable ai, linguistic profiling, natural language processing, text classification

## Abstract

Background

Emotion recognition in text underpins a wide range of human-centered artificial intelligence (AI) applications, yet existing frameworks either prioritize accuracy at the expense of interpretability or analyze emotional language through a single lens. This study developed and evaluated the Multi-layer Emotion Linguistic Profiling Framework (MELPF), a reproducible protocol integrating lexical, structural, and model-level interpretability analyses to characterize how emotional meaning is encoded in short text.

Methods

The Kaggle Emotions Dataset (16,000 training, 2,000 validations, and 2,000 test instances; six emotion categories) was used. The corpus exhibits pronounced class imbalance: joy (33.5%) and sadness (29.2%) dominate, while surprise is underrepresented (3.6%); in addition, all statistics were derived strictly from training instances to prevent data leakage. MELPF operates across five stages: preprocessing; lexical profiling (unigram/bigram distributions, term frequency-inverse document frequency {TF-IDF}, pointwise mutual information (PMI), and co-occurrence analysis); structural profiling (part-of-speech {POS} distribution and template identification); supervised classification using logistic regression, a linear support vector machine (SVM), random forest, a bidirectional long short-term memory (BiLSTM) network, and Bidirectional Encoder Representations from Transformers (BERT) (base-uncased variant); and cross-layer synthesis via feature-importance mapping and attention visualization.

Results

BERT achieved the highest macro-F1 (the macro-averaged F1 score, i.e., the harmonic mean of precision and recall; 0.92, accuracy 0.93), followed by BiLSTM (0.89), linear SVM (0.88), logistic regression (0.86), and random forest (0.81). Ablation confirmed that PMI-derived and POS-based features improved macro-F1 from 0.88 to 0.89 over the TF-IDF baseline. Lexical profiling identified category-discriminative unigrams and high-PMI bigrams, while structural profiling revealed a dominant first-person copular frame. Feature-importance scores and attention visualization confirmed alignment between salient tokens and model decisions.

Conclusion

MELPF offers a transparent, reproducible methodology for examining emotional meaning across multiple linguistic layers, bridging theoretical linguistics and applied NLP.

## Introduction

Recognizing human emotion from written language has become a central challenge in natural language processing (NLP) as digital communication increasingly replaces face-to-face interaction. Text carries nuanced affective signals through word choice, phrasing, and grammatical structure, and computational systems capable of detecting these signals are essential to applications ranging from mental-health monitoring and customer-feedback analysis to empathetic conversational agents and crisis-detection tools [1,2]. The breadth of these applications has motivated substantial investment in emotion recognition, producing a field that has progressed from rule-based lexicons through classical machine learning to deep transformer architectures [1,3].

Despite this progress, two structural limitations constrain the current state of the art. First, high-performing models, particularly deep-learning and transformer variants, tend to function as opaque systems whose internal decision mechanisms are difficult to interpret [3,4]. This opacity limits deployment in accountability-sensitive settings and obscures the linguistic reasoning that underpins model predictions. Second, existing studies characteristically examine emotional language through a single analytical lens: Lexical approaches map affective vocabulary but ignore grammatical structure, syntactic approaches capture framing but overlook distributional lexical patterns, and deep-learning approaches model context without explicitly representing linguistic structure [1,5]. The resulting fragmentation means that no prior framework has systematically examined how lexical, structural, and contextual cues interact to constitute emotional meaning in short text.

These limitations motivate the present study, which introduces the Multi-layer Emotion Linguistic Profiling Framework (MELPF). MELPF is not a new classification architecture; it is a structured, reproducible analytical protocol that operationalizes multi-layer linguistic profiling, spanning unigram and bigram frequency, pointwise mutual information (PMI), part-of-speech (POS) distribution, and contextual embedding, while linking each profiling layer to the decision patterns of classical and transformer-based models through interpretability mechanisms. The choice of linguistic profiling as the organizing principle is grounded in evidence that short emotional text exhibits highly regular lexical and syntactic structures that both reveal and constrain emotional meaning [1,5,6]. By making these structures explicit and connecting them to model behavior, MELPF addresses the interpretability deficit in contemporary emotion recognition.

The framework was applied to the Kaggle Emotions Dataset (16,000 training instances; six-category labels), a corpus of short emotional statements exhibiting the compact lexical and syntactic patterns characteristic of social-media and conversational text. This dataset was selected for its publicly accessible splits, its six-category granularity, and its alignment with the study's focus on short-form emotional expression. Its limitations, single-domain provenance, class imbalance, and the absence of conversational context, are explicitly acknowledged and addressed in the Strengths, limitations, and future work section.

The main objective of this study was to develop and evaluate MELPF as a structured, reproducible analytical protocol capable of characterizing how emotional meaning is encoded in short text across multiple linguistic layers while maintaining alignment with model-level interpretability evidence. Three specific research questions (RQs) guided the study. RQ1 asked whether the integration of multi-layer linguistic profiling, spanning lexical frequency, PMI-based associations, and POS-based structural features, provides diagnostic insight into emotion classification errors beyond what standard term frequency-inverse document frequency (TF-IDF) baselines offer. RQ2 asked to what extent transformer attention patterns converge with linguistically salient tokens identified through MELPF profiling, thereby assessing the degree of alignment between computational and linguistic representations of emotional meaning. RQ3 asked which linguistic profiling layer contributes most to explaining systematic misclassification between closely related emotion categories, with particular focus on the sadness-fear and joy-love pairs.

The primary contributions of the study are (i) the development of MELPF as a reproducible five-stage protocol for multi-layer linguistic profiling of short emotional text, (ii) empirical demonstration that PMI-based and POS-based profiling layers provide complementary diagnostic information beyond TF-IDF baselines, and (iii) the systematic alignment of linguistic profiling outputs with model-level interpretability evidence from feature-importance mapping and transformer attention visualization.

Related work

Lexicon-Based Foundations

Early textual emotion analysis relied on affective lexicons that associated individual tokens with predefined emotional categories. Domain-specific resources strengthened performance by capturing context-sensitive vocabulary absent from generic sentiment databases [7,8]. These approaches established the centrality of explicit affective vocabulary in short emotional text and continue to inform modern frameworks, including MELPF's lexical profiling layer.

Compositional and Multiword Lexical Patterns

Research subsequently demonstrated that emotional meaning frequently emerges from multiword units rather than isolated tokens. Compact bigram frames such as feel sad and am happy recur systematically across emotion categories and outperform unigram features for fine-grained classification [9,10]. PMI-based analyses have quantified the strength of these associations, providing statistically grounded alternatives to frequency counts alone [11].

Structural Linguistic Features

Part-of-speech analyses revealed that emotional statements commonly adopt first-person copular constructions (I feel ___; I am ___), indicating that emotional expression in short text is not only lexically anchored but also syntactically constrained [7,12]. Structural regularity of this kind makes POS-based features informative for classification and interpretable for analysis, motivating their inclusion as a distinct profiling layer in MELPF.

Classical Machine Learning

Support vector machines (SVMs), logistic regression, and random forests with engineered feature sets (term frequency-inverse document frequency {TF-IDF}, n-grams, and syntactic markers) formed a critical methodological baseline, establishing that feature quality substantially influences performance and that lexical-structural combinations outperform single-layer representations [13]. These models also provide transparent feature-importance scores, which MELPF exploits for interpretability.

Deep Learning and Transformer Architectures

Bidirectional long short-term memory (BiLSTM) networks enabled the sequential modelling of emotional cues without explicit feature engineering, improving performance on longer or more contextually complex texts [14]. Transformer architectures, led by Bidirectional Encoder Representations from Transformers (BERT), subsequently achieved state-of-the-art results across multiple emotion datasets by capturing global contextual dependencies through multi-head attention [3,15-17]. However, the relationship between attention weights and model decisions remains contested, and attention alone does not constitute a complete explanation of model behavior [18].

Interpretability in Emotion Recognition

The NLP interpretability literature underscores the difficulty of translating internal model computations into human-interpretable rationales [18]. Feature-importance approaches offer one complement to attention visualization, but neither mechanism alone provides a complete account of transformer decision processes [4]. MELPF addresses this limitation by combining both approaches and anchoring their outputs to linguistically grounded profiling evidence.

Dataset Reliability and Contextual Variability

Research on emotion dataset quality has shown that emotional labels in short text are sensitive to missing conversational context, generating annotator disagreement when cues are sparse or ambiguous [19]. This finding is directly relevant to the Kaggle dataset, whose single-sentence items lack discourse context. The Strengths, limitations, and future work section addresses the consequences for generalization.

Research Gap

Taken together, these contributions reveal a consistent methodological gap: no prior framework systematically examines lexical, structural, and contextual layers jointly and links each layer to model interpretability evidence. Individual studies address one or two of these dimensions, but their siloed designs preclude a unified understanding of how linguistic cues collectively shape emotional meaning and influence classification outcomes. MELPF fills this gap by providing a five-stage reproducible protocol that integrates all three analytical dimensions and operationalizes their diagnostic utility through ablation analysis and cross-layer synthesis [13,20].

## Materials and methods

Study design and reporting

This study is a retrospective, computational, secondary data analysis of a pre-existing public text corpus, rather than a prospectively recruited clinical, survey-based, or observational human-subject investigation. It applies the Multi-layer Emotion Linguistic Profiling Framework (MELPF) protocol to the Kaggle Emotions Dataset and does not involve the collection of new data from human participants; the unit of analysis is the individual short-text instance, not an individual person, and no participants were recruited, contacted, or observed by the study team.

Sampling Technique

The Kaggle Emotions Dataset was used in its entirety as released by its original curators, with the predefined training, validation, and test partition (16,000/2,000/2,000 instances) retained unmodified; no additional resampling, oversampling, or under-sampling of the six emotion categories was performed, so the class imbalance reported below reflects the corpus as distributed. Labels were assigned by the dataset's original curators through an automated labelling procedure prior to public release (see dataset limitations below); the present study did not perform de novo manual annotation of the text instances.

Inclusion/Exclusion Criteria

All instances contained within the official training, validation, and test splits were included, provided they carried exactly one of the six defined emotion labels (anger, fear, joy, love, sadness, surprise) and were non-empty after preprocessing. No instance met an exclusion criterion in the released version of the dataset used: none were removed for length, duplication, or missing text, since the splits as obtained from Kaggle did not contain such artefacts.

Confounders

Three methodological confounders were identified and addressed. Pronounced class imbalance across the six emotion categories was addressed by reporting macro-averaged rather than micro-averaged performance metrics throughout so that minority categories such as surprise are not numerically overwhelmed by majority categories such as joy. Single-domain provenance, the corpus consists exclusively of short, social-media-style statements, was addressed by explicitly restricting generalization claims to this register and discussing it in the Strengths, limitations, and future work section. Potential label noise arising from the dataset's automated labelling procedure could not be corrected within the scope of this study and is likewise acknowledged as a limitation rather than treated as resolved.

Location

As a computational study conducted entirely on a publicly available, pre-existing dataset, no physical study site, clinic, or participant-recruitment location applies. All analyses were performed by the authors at their respective institutions: King Abdullah Medical Complex, Jeddah, Saudi Arabia; Research Assistant (RA) Company for Research and Studies, Riyadh, Saudi Arabia; and Pioneers Academy, Amman, Jordan.

Study Duration

All experiments, analyses, and manuscript preparation for this study were conducted between October 2025 and December 2025.

Sample-Size Calculation

Because the Kaggle Emotions Dataset is a fixed, publicly released benchmark whose training, validation, and test partitions were determined a priori by its original curators rather than by the present authors, no prospective sample-size or power calculation was performed. The available sample (20,000 instances in total) exceeds the sample sizes typically used in comparable published emotion-classification benchmarks, and macro-averaged evaluation was adopted specifically to reduce the influence of the smallest category (surprise, n = 572 training instances) on the overall performance estimates.

Statistical Tests Used

Model comparisons in this study are reported as single point estimates, accuracy, precision, recall, and macro-F1, computed once on the held-out test set for each model, following common practice in the emotion-classification literature; formal inferential statistical testing (e.g., paired significance tests or bootstrap confidence intervals) was not performed to compare between models, and differences between the reported metrics should therefore be interpreted descriptively rather than as tests of statistical significance. Fivefold cross-validation grid search was used for classical-model hyperparameter selection only (described under supervised classification, below); this procedure was used for model tuning and not as an inferential test.

Institutional Review Board (IRB)/Institutional Ethics Committee​​​​​​​ (IEC) Details

This study analyzed a publicly available, fully anonymized, pre-existing dataset that does not permit the identification of individual contributors and did not involve direct interaction with, or recruitment of, human participants by the authors. Consistent with institutional guidelines for secondary analyses of de-identified public data, formal review by an Institutional Review Board (IRB) or Institutional Ethics Committee (IEC) was therefore not required for this study; this determination is elaborated further under ethics approval in the Human subjects section.

Dataset

The Kaggle Emotions Dataset comprises 20,000 short emotional statements partitioned into fixed splits: 16,000 training, 2,000 validation, and 2,000 test instances. Each statement is labelled with one of six emotion categories. The dataset exhibits pronounced class imbalance: in the training split, joy accounts for 5,362 instances (33.5%), sadness for 4,666 (29.2%), anger for 2,159 (13.5%), fear for 1,937 (12.1%), love for 1,304 (8.2%), and surprise for 572 (3.6%). This imbalance is maintained proportionally across the validation and test splits (Table 1 reports full counts). Macro-averaged metrics were used throughout to provide equal weighting across categories regardless of support size.

**Table 1:** Class distribution across dataset splits N and n denote absolute instance counts for the training and validation/test splits, respectively; % denotes the percentage of the corresponding split total. Splits were fixed by the dataset's original curators and are reported for descriptive purposes only; because the validation and test splits are subsets of the same predefined partition rather than independent samples, no significance test was applied to compare the distributions across splits

Emotion	Training		Validation		Testing	
	N	%	n	%	n	%
Joy	5,362	33.5	704	35.2	695	34.8
Sadness	4,666	29.2	550	27.5	581	29.1
Anger	2,159	13.5	275	13.8	275	13.8
Fear	1,937	12.1	212	10.6	224	11.2
Love	1,304	8.2	178	8.9	159	8.0
Surprise	572	3.6	81	4.1	66	3.3
Total	16,000	100	2,000	100	2,000	100

Dataset limitations are acknowledged explicitly: the corpus is drawn from a single online domain (short social-media-style statements), lacks conversational context, and was compiled through automated labelling. These characteristics constrain generalization to other genres (narratives, dialogues, and clinical notes).

Preprocessing Pipeline

A standardized preprocessing workflow was applied to all splits. Critically, all statistics derived from preprocessing (TF-IDF weights, PMI values, co-occurrence counts, and vocabulary indices) were computed exclusively on training-set instances and applied to the validation and test sets without refitting, thereby preventing data leakage [21]. The following steps were applied in sequence.

All characters were first converted to lower case. Tokenization followed: whitespace tokenization was applied for the classical and BiLSTM models, whereas BERT used its native WordPiece tokenizer (BERT-base-uncased vocabulary), which handles sub-word segmentation and preserves affective morphemes (e.g., afraid → afraid; fearful → fear + ##ful); no additional tokenization was applied to BERT inputs to avoid double processing. All punctuation was then removed except exclamation marks, which carry affective salience in short emotional text [6]. The WordNet lemmatizer (Natural Language Toolkit {NLTK} 3.8.1) was applied to the classical-model and BiLSTM inputs only; lemmatization reduces surface variation (feeling → feel, worried → worry) while preserving the root lexical form that carries emotional information, and it was not applied to BERT inputs because BERT's contextual embeddings already handle morphological variation. Stopword removal was applied only for the lexical statistics (unigram/bigram counts, PMI), not for model-training inputs, to prevent the removal of function words that contribute to syntactic framing (e.g., the copular structure I am ___ depends on the pronoun and auxiliary). Finally, the six emotion categories were label-encoded as integers 0-5.

MELPF: Framework Design and Rationale

MELPF is designed as a structured analytical protocol rather than a novel classification architecture. Its five-stage sequential design is motivated by the principle that interpretable emotion analysis requires the explicit examination of linguistic structure before, and alongside, computational modelling. The framework is reproducible: each stage produces intermediate artefacts (frequency tables, PMI rankings, POS distributions, model weights, and attention matrices) that are retained and compared across stages.

The choice of linguistic profiling as the organizing principle rests on three empirical observations from the literature: (a) Short emotional text exhibits highly regular lexical and syntactic structures that constrain emotional meaning [1,5], (b) these structures are partially recoverable from distributional statistics without requiring deep semantic parsing [9,11], and (c) aligning profiling outputs with model interpretability evidence provides a principled bridge between theoretical linguistics and applied NLP [1,3,18].

The choice of BERT and BiLSTM as the deep-learning components is motivated by the characteristics of the dataset. BiLSTM is appropriate for short sequential text where positional information and moderate-range dependencies matter and where computational cost must be controlled for reproducibility. BERT-base-uncased is well-suited to the informal, short-sentence register of the dataset: its WordPiece vocabulary handles colloquial forms, and its 512-token context window comfortably accommodates the dataset's short instances (mean length: approximately 12 tokens). Both architectures have been validated on comparable emotion-recognition tasks, and their complementary designs, sequential versus contextual, allow meaningful architectural comparison within MELPF [14-16]. The five stages are described below.

Stage 1: Preprocessing

Preprocessing followed the standardized workflow described above under Preprocessing Pipeline. This workflow was applied identically and independently across the training, validation, and test splits to preserve methodological consistency across all downstream analytical stages, consistent with the data-leakage-prevention procedure outlined above.

Stage 2: Lexical Profiling

Lexical profiling characterized the distribution of words and multiword expressions across emotion categories using four complementary methods. Unigram and bigram frequency counts were computed over the preprocessed training corpus, and the top-N items per emotion category were identified for comparative analysis. Term frequency-inverse document frequency (TF-IDF) weighting was then applied using scikit-learn 1.4.0's TfidfVectorizer, with the fit restricted to the training set; inverse-document-frequency values were computed on training instances only and applied to the validation and test sets at inference time.

Pointwise mutual information (PMI) was computed for adjacent bigrams as PMI(w1, w2 | emotion) = log[P(w1,w2|emotion) / (P(w1|emotion) × P(w2|emotion))], with values restricted to bigrams occurring at least five times in the training set within a given emotion class to exclude unreliable low-frequency associations, a threshold selected in line with standard practice for collocation analysis [11]. PMI quantifies the degree to which two words co-occur more than chance within an emotion category, providing a statistically grounded complement to raw frequency counts. Finally, co-occurrence counts were computed over a two-token sliding window on training-set instances, with all statistics fit on the training set only.

Stage 3: Structural Linguistic Profiling

Structural profiling examined part-of-speech (POS) patterns using spaCy 3.6 (en_core_web_sm model), analyzing two features. The first was POS distribution: the proportional distribution of nouns (NOUN), verbs (VERB), adjectives (ADJ), and pronouns (PRON) across emotion categories. The second was POS template identification: High-frequency trigram POS sequences (e.g., PRON-AUX-ADJ and PRON-VERB-ADV) were identified to characterize the grammatical framing typical of each emotion category, and templates were ranked by frequency and inspected for category specificity.

Structural profiling was limited to POS-level analysis to maintain alignment with the study's analytical scope and to ensure reproducibility without requiring dependency-parsing infrastructure. POS tags were assigned by spaCy's rule-based tagger, and all annotations were derived from training-set instances only.

Stage 4: Supervised Classification

Classical models: Three classical classifiers were implemented using scikit-learn 1.4.0 (Python 3.10.12). Logistic regression was configured with L2 regularization, with the regularization parameter C selected via fivefold cross-validation grid search over {0.01, 0.1, 1, 10}, solver = lbfgs, max_iter = 1000, and random_state = 42. The linear support vector machine used the LinearSVC implementation, with C likewise selected via fivefold CV grid search over {0.01, 0.1, 1, 10}, max_iter = 2000, and random_state = 42. The random forest classifier used n_estimators = 500, with max_depth selected from {10, 20, None} via fivefold CV, min_samples_split = 5, and random_state = 42. All three classical models used the same feature set: TF-IDF vectors (unigram + bigram, max_features = 50,000) fit on the training data only.

Deep-learning models: The BiLSTM model was implemented in TensorFlow 2.13.0 using Keras. Its architecture used 100-dimensional pre-trained Global Vectors for Word Representation (GloVe) embeddings (glove.6B.100d), a bidirectional long short-term memory (LSTM) layer with 64 units per direction, a dropout rate of 0.3, and a dense output layer with softmax activation. The model was trained using the Adam optimizer with a learning rate of 1e-3 and a batch size of 64, for a maximum of 20 epochs with early stopping (patience = 3, monitored on validation macro-F1). The random seed was fixed at 42 (random_state / tf.random.set_seed = 42).

BERT-base-uncased: The BERT-base-uncased model was implemented using HuggingFace Transformers version 4.38.2. Fine-tuning was performed using the AdamW optimizer with a learning rate of 2e-5 and a linear warm-up over 10% of the training steps, a batch size of 32, and five training epochs. The maximum sequence length was set to 64 tokens, which covered more than 99% of instances at this length, and the random seed was fixed at 42. Attention weights were extracted from the final encoder layer for the interpretability analysis.

Stage 5: Cross-Layer Synthesis and Interpretability

The final MELPF stage integrates outputs across all preceding layers using three complementary techniques. Feature-importance mapping used logistic regression coefficient magnitudes and linear SVM weight vectors to identify the lexical tokens with the highest discriminative weight per emotion category. Attention visualization used BERT's final-layer, mean-pooled attention weights, computed for a stratified sample of 50 correctly classified and 50 misclassified instances per emotion category, with attention scores averaged across attention heads and normalized per instance. Finally, cross-layer alignment analysis compared tokens ranked highly by feature-importance mapping with tokens receiving high attention weights, cross-referencing both against the top-PMI bigrams and POS-profile findings to assess convergence across linguistic and computational representations. This synthesis directly addresses the interpretability deficit in prior work by providing a principled, evidence-grounded account of which linguistic features drive classification, rather than relying on attention visualization alone.

Ablation study design: To quantify the diagnostic contribution of each MELPF layer (RQ1 and RQ3), an ablation-based evaluation was conducted using the linear SVM classifier (selected for its transparent feature-importance interpretation and strong baseline performance). Three configurations were compared: Configuration A, the baseline, used TF-IDF features only (unigram + bigram). Configuration B added TF-IDF plus PMI-derived lexical association features, specifically, the top-50 PMI bigrams per emotion category, represented as a binary presence indicator. Configuration C extended Configuration B with POS distribution features, namely, the relative frequency of NOUN, VERB, ADJ, and PRON per instance.

Performance was evaluated using macro-F1 and per-class confusion matrices on the test set. Per-class F1 for commonly confused pairs (sadness versus fear; joy versus love) was examined specifically to address RQ3.

## Results

Results are organized to align with the five MELPF stages and the three research questions: lexical profiling, PMI-based associations, co-occurrence structure, structural linguistic profiling, model performance, and ablation and cross-layer synthesis.

Lexical profiling

Unigram profiling following stopword removal revealed two distinct strata of lexical variation across the six emotion categories. A shared stratum, comprising feel, feeling, like, and im, appeared in the top-five unigrams of every category, reflecting the pervasive use of introspective first-person frames in short emotional text. A category-discriminative stratum provided the basis for emotion differentiation: Anger and sadness shared the truncated token t (contracted from don't/can't), reflecting negation-heavy constructions; love featured the token love as a top-five unigram absent from other categories; and surprise contained amazed, the sole category-exclusive evaluative term in the top-five rankings (Table 2).

**Table 2:** Top-five unigrams per emotion category (after stopword removal) This table presents the top-five unigrams for each emotion class after removing function words. Emotional markers ("feel" and "feeling") were preserved due to their semantic importance. The distribution shows strong prototypical cues across emotions, with "feel/feeling" and interpersonal markers ("like" and "im") dominating short-form emotional text. Rankings are based on raw frequency counts within the training split only, expressed as ordinal rank rather than a normalized rate; no significance testing was applied to the word-frequency differences shown

Emotion	Top Unigrams (Frequency Rank 1-5)
Anger	feel, feeling, like, im, t
Fear	feel, feeling, im, like, little
Joy	feel, feeling, like, im, really
Love	feel, feeling, like, im, love
Sadness	feel, feeling, like, im, t
Surprise	feel, feeling, like, im, amazed

The concentration of shared tokens in top-five rankings indicates that unigram frequency alone provides limited discriminative power; category-specific vocabulary becomes visible only at lower-frequency ranks or when filtered by TF-IDF weighting, which down-weights ubiquitous terms. Full unigram rankings (top 20 per category) are provided in the Appendices.

PMI-based emotion-word associations

PMI analysis identified high-association bigrams that provide stronger category discrimination than unigram frequency counts. For anger, high-PMI bigrams included mad at and get mad, reflecting direct frustration expression. Sadness featured never felt and bit miserable, indicating lexical frames associated with prolonged or evaluated negative affect. Fear displayed very anxious and be afraid, constructions centered on apprehension. Joy produced gut feeling, thankful for, and excited about, indicating diverse positive evaluative frames. Love was characterized by loved by and feeling generous, reflecting relational and prosocial expression. Surprise yielded pleasantly surprised and amazed at, associated with evaluating unexpected events positively (Table 3).

**Table 3:** High-PMI bigrams per emotion category (frequency threshold ≥ 5, training set only) PMI values were computed on training-set instances only and were restricted to bigrams occurring at least five times within a given emotion category (frequency threshold ≥5, as stated in the table title), following standard practice for collocation analysis [11]; bigrams are listed in descending order of PMI score. PMI is a unitless log-ratio statistic, and no formal significance threshold (e.g., a p-value cutoff) was applied, consistent with its use here as an exploratory, descriptive measure rather than an inferential test PMI: pointwise mutual information

Emotion	High-PMI Bigrams (Examples)
Anger	stopped feeling, mad at, get mad, feeling particularly, remember feeling
Fear	fearful or, very anxious, remember feeling, be afraid, without feeling
Joy	gut feeling, id love, thankful for, excited about, feeling fairly
Love	loved by, feeling generous, feeling particularly, m feeling, feeling quite
Sadness	mad at, never felt, feeling particularly, be happy, bit miserable
Surprise	pleasantly surprised, remember feeling, surprised by, amazed at, feeling pretty

Notably, remember feeling appeared as a high-PMI bigram across multiple categories (anger, fear, surprise), indicating a broadly used retrospective emotional frame whose category-specific interpretation depends on adjacent lexical context, a finding that underscores the value of contextual embedding approaches for cross-category disambiguation. Full PMI rankings and scores are provided in the Appendices.

Lexical co-occurrence structure

Co-occurrence analysis over a two-token window identified stable lexical templates for each category. All categories exhibited the dominant frame (feel, ___) anchored by the stative verb feel, confirming that self-referential introspective constructions constitute the primary expressive structure across categories. Category-specific secondary frames included (feel, afraid) and (feel, anxious) for fear, (feel, angry) and (mad, at) for anger, (feel, happy) and (feel, joyful) for joy, (love, you) and (feel, loved) for love, (feel, sad) and (feel, down) for sadness, and (feel, surprised) and (amazed, by) for surprise. These template structures indicate that emotional differentiation in the dataset depends primarily on the affective descriptor in the second position of the copular frame, rather than on variation in the framing verb itself (Table 4).

**Table 4:** Representative co-occurrence clusters per emotion category Co-occurrence counts were computed over a two-token sliding window on training-set instances only and are ranked by raw co-occurrence frequency (a unitless count, not a percentage or rate); no significance testing was applied, as the pairs shown are the most frequent co-occurrences per category rather than the output of a hypothesis test

Emotion	Dominant Co-occurrence Pairs
Anger	(feel, angry), (mad, at), (get, mad), (angry, about)
Fear	(feel, afraid), (feel, anxious), (be, afraid), (feel, nervous)
Joy	(feel, happy), (feeling, great), (happy, today), (feel, joyful)
Love	(love, you), (feel, loved), (feeling, blessed), (love, this)
Sadness	(feel, sad), (feel, down), (feeling, low), (never, felt)
Surprise	(feel, surprised), (amazed, by), (surprised, by), (feeling, amazed)

Structural linguistic profiling

POS distribution analysis revealed that pronoun density was highest in the joy and love categories (reflecting personal, relational expression), while adjective density was highest in surprise (reflecting evaluative novelty terms such as amazed and astonished). Verb density was relatively uniform across categories, consistent with the dominance of the copular frame. Across all six categories, the pronoun-auxiliary-adjective trigram template (PRON-AUX-ADJ; e.g., I am happy; I was shocked) was the most frequent POS sequence, appearing in 38%-52% of instances depending on category. The pronoun-verb-adverb template (PRON-VERB-ADV; e.g., I feel really) was the second most frequent across joy and love. These structural regularities confirm that emotion expression in this dataset is syntactically stereotyped, which both aids classification, because structural cues provide complementary information to lexical features, and constrains generalization to genres with greater syntactic diversity.

Model performance

Table 5 reports macro-averaged performance metrics on the test set. BERT achieved the highest overall performance (macro-F1 = 0.92, accuracy = 0.93), consistent with transformer superiority on contextualized short-text classification tasks [15,16]. BiLSTM performed strongly (macro-F1 = 0.89), exceeding all classical baselines. Among classical models, linear SVM achieved the highest macro-F1 (0.88), followed by logistic regression (0.86) and random forest (0.81). The comparatively weaker random forest performance reflects known limitations of tree-based methods on high-dimensional sparse text representations [13]. All models struggled most with the surprise category (smallest class, n = 66 test instances), and confusion between sadness and fear was the most frequent cross-category error in classical models, motivating the targeted ablation analysis in the next section.

**Table 5:** Performance of machine-learning and deep-learning models on the test set (macro-averaged) All metrics are macro-averaged across six emotion categories on the held-out test set. Full per-class precision, recall, and F1 are provided in the Appendices. Accuracy, precision, recall, and macro-F1 are unitless proportions ranging from 0 to 1 (equivalently 0%-100%) and were computed once, as point estimates, on the single 2,000-instance held-out test set; no formal inferential statistical test (e.g., a paired significance test) was used to compare models, so the differences between rows should be interpreted descriptively rather than as tests of statistical significance SVM, support vector machine; BiLSTM, bidirectional long short-term memory; BERT, Bidirectional Encoder Representations from Transformers

Model	Accuracy	Precision	Recall	Macro-F1
Logistic regression	0.87	0.86	0.87	0.86
Linear SVM	0.89	0.88	0.89	0.88
Random forest	0.82	0.81	0.81	0.81
BiLSTM	0.90	0.89	0.90	0.89
BERT (base and uncased)	0.93	0.92	0.93	0.92

Ablation analysis and cross-layer synthesis

Table 6 reports the macro-F1 of the linear SVM under the three ablation configurations. Adding PMI-based lexical features to the TF-IDF baseline (Configuration B) preserved performance at 0.88, indicating that the PMI features do not conflict with or duplicate TF-IDF signals. Adding POS-based structural features (Configuration C) produced a consistent improvement to macro-F1 = 0.89.

**Table 6:** Diagnostic contribution of linguistic profiling layers to error explanation Macro-F1 is a unitless proportion ranging from 0 to 1 (equivalently 0%-100%), computed once for the linear support vector machine (SVM) classifier on the 2,000-instance held-out test set under each feature configuration. No formal inferential statistical test (e.g., a paired significance test) was applied to the differences between configurations; values should be interpreted as descriptive point estimates, consistent with the Statistical Tests Used section description in the Materials & Methods PMI, pointwise mutual information; POS, part-of-speech; TF-IDF, term frequency-inverse document frequency

Configuration	Features	Macro-F1
A: baseline	TF-IDF only (unigram + bigram)	0.88
B: + PMI	TF-IDF + PMI-derived bigram features	0.88
C: + PMI + POS	TF-IDF + PMI features + POS distribution features	0.89

Regarding RQ3, sadness versus fear confusion decreased from 14.2% of sadness instances misclassified as fear (Configuration A) to 11.8% (Configuration C), representing a 2.4 percentage-point reduction. This improvement aligns with the PMI analysis showing distinct high-PMI bigrams for fear (very anxious, be afraid) versus sadness (never felt, bit miserable), constructions that are not well-captured by raw frequency features but are highlighted by PMI weighting. Joy versus love confusion was less affected by profiling layers (3.1% → 2.9%), consistent with the high lexical and structural overlap between these categories identified in the lexical, syntactic, and semantic profiling analyses presented above.

Cross-layer alignment analysis confirmed strong convergence between interpretability outputs and profiling findings. Feature-importance scores from logistic regression assigned the highest weights to the same tokens that dominated PMI rankings within each category (e.g., amazed for surprise, afraid for fear, angry for anger). BERT attention weights concentrated on affective descriptors (adjectives and affective verbs) and were distributed over the first-person frame (I, am, feel), consistent with the POS template analysis. Attention was notably more diffuse for commonly confused pairs (sadness/fear), reflecting the smaller number of strongly discriminative tokens in these categories, a finding that aligns with the PMI evidence showing overlapping high-PMI bigrams (e.g., remember feeling, feeling particularly) across these two categories.

## Discussion

Summary of principal findings

This study developed MELPF, a five-stage linguistic profiling framework integrating lexical, structural, and model-level interpretability analyses for emotion recognition in short text. Applied to the Kaggle Emotions Dataset, MELPF demonstrated that (i) emotional expression is systematically structured around a dominant first-person copular frame whose discriminative content resides in the affective descriptor, (ii) PMI-based lexical features and POS-based structural features provide complementary diagnostic information beyond TF-IDF baselines, (iii) transformer models achieve the highest predictive accuracy and their attention patterns align with the linguistically salient tokens identified through MELPF profiling, and (iv) cross-layer synthesis reveals that sadness-fear misclassification is driven by shared high-PMI bigrams (remember feeling, feeling particularly), while joy-love confusion reflects deep lexical and structural overlap that current profiling layers cannot fully resolve.

Interpretation of lexical and PMI findings

The concentration of shared tokens (feel, feeling, im) across all six categories indicates that unigram frequency is a weak discriminator in this dataset. This finding reinforces prior work demonstrating that emotional vocabulary in short online text is compact and formulaic and motivates the PMI and co-occurrence analyses as necessary complements [1,5,22]. The cross-category PMI bigram remember feeling exemplifies a lexically underspecified emotional frame: its category assignment depends entirely on lexical and discourse context unavailable from the bigram itself. This pattern has practical implications for classification: models that rely on surface n-gram statistics will assign such frames inconsistently, while contextual models (BERT) can leverage surrounding tokens to resolve the ambiguity, consistent with BERT's superior performance on the confused categories [15].

Structural regularities and their implications

The dominance of the PRON-AUX-ADJ template (I am happy; I was shocked) across all six categories reflects the dataset's origin in self-report emotional statements [5,7]. This structural stereotyping has two implications. Analytically, it means that POS features provide limited within-dataset discrimination beyond what lexical features already capture, explaining the modest ablation gain from POS inclusion (0.88 → 0.89). Practically, it raises a generalization concern: models trained on this syntactically uniform corpus may underperform on emotionally rich but structurally diverse genres such as literary narrative, clinical case descriptions, or argumentative discourse. This is addressed in the Strengths, limitations, and future work section.

Model performance and architecture alignment

BERT's superiority over BiLSTM and classical models aligns with the established advantage of contextual embeddings for short-text classification [15,16,23]. The architecture is well-matched to the dataset: the dataset's short sequences (mean ≈ 12 tokens) fall well within BERT's context window, and the informal register is well-represented in the BERT-base-uncased pre-training corpus. BiLSTM's strong performance relative to classical models confirms that the sequential modelling of positional information provides value even in short text. The random forest's relative weakness reflects the sparse-vector representation challenge [24] and is consistent with prior comparative evaluations on the same dataset type [13].

Interpretability alignment and practical value

The cross-layer alignment between feature-importance scores, attention weights, and PMI rankings constitutes the principal interpretability contribution of MELPF. This convergence demonstrates that transformer models prioritize the same linguistically motivated tokens that human-interpretable profiling identifies as emotionally salient [15,25]. This finding has direct practical value: it means that the MELPF profiling layers can function as an independent diagnostic audit of model behavior, identifying which token types drive predictions and whether those tokens correspond to interpretable emotional constructs or to dataset artefacts. The diffuse attention observed for sadness-fear confusion suggests the absence of strongly discriminating features for these categories in the current lexical representation, pointing to a concrete engineering direction: augmenting these categories with affect-specific features (e.g., valence and arousal scores from affective lexicons) may improve disambiguation [8].

Strengths, limitations, and future work

MELPF's principal strength is its integration of multiple analytical perspectives into a reproducible protocol. By explicitly preventing data leakage, reporting implementation details (library versions, hyperparameters, and random seeds), and conducting a targeted ablation study, the study addresses the key methodological reproducibility standards expected of computational NLP research.

Three limitations are acknowledged. First, the study relies on a single dataset from one domain (short social-media-style statements), limiting generalization to other genres. The syntactic stereotyping documented in the Structural linguistic profiling section means that MELPF's structural profiling layer is likely more informative in datasets with greater grammatical diversity. Second, attention visualization is used as a complementary rather than primary interpretability mechanism, consistent with evidence that attention weights alone do not reliably reflect model decision processes [18]. Third, the class imbalance (particularly for surprise, n = 572 training instances) means that per-class results for this category should be interpreted with caution.

Future extensions should apply MELPF to multilingual corpora, conversational datasets, and domain-specific emotional text (e.g., clinical records and social-support forums) to test the generalizability of its profiling patterns and assess whether the five-stage protocol requires adaptation for different genres. Incorporating affective lexicon features (valence, arousal, and dominance scores) as an additional profiling layer may enhance sadness-fear discrimination. Finally, applying MELPF to datasets with richer contextual annotations (e.g., GoEmotions) would enable testing of whether discourse-level features add diagnostic value beyond the sentence-level profiling conducted here [26].

## Conclusions

This study introduced MELPF, a reproducible five-stage analytical framework designed to characterize emotional expression in short text through integrated lexical profiling, structural linguistic analysis, and model-level interpretability. Applied to the Kaggle Emotions Dataset, MELPF demonstrated that short emotional text is organized around a dominant first-person copular frame, that PMI-based and POS-based profiling layers provide complementary diagnostic information beyond TF-IDF baselines, and that transformer attention patterns converge with linguistically motivated token rankings. BERT achieved the strongest predictive performance (macro-F1 = 0.92), and cross-layer synthesis identified shared PMI bigrams as the principal driver of sadness-fear misclassification. The framework's contribution lies in its methodological integration rather than in any individual technique: by anchoring computational predictions to explicit linguistic evidence at multiple levels of analysis, MELPF provides a principled pathway toward emotion-recognition systems that are simultaneously accurate, interpretable, and diagnostically informative. The protocol is designed to be reproducible and extensible, and its application to diverse datasets and genres represents a clear priority for future research.

## Appendices

Table 7 shows the full unigram rankings (top 20 per category). 

**Table 7:** Top-20 unigrams per emotion (real data)

Emotion	Unigram	Frequency
sadness	i	9,440
sadness	feel	4,079
sadness	and	3,372
sadness	to	2,935
sadness	the	2,682
sadness	a	2,051
sadness	feeling	1,913
sadness	of	1,762
sadness	that	1,625
sadness	my	1,556
sadness	in	1,125
sadness	it	1,101
sadness	like	1,064
sadness	so	1,016
sadness	for	962
sadness	im	875
sadness	was	837
sadness	me	828
sadness	but	818
sadness	have	777
anger	i	4,484
anger	feel	1,796
anger	and	1,584
anger	to	1,449
anger	the	1,403
anger	a	1,000
anger	feeling	918
anger	that	852
anger	of	798
anger	my	709
anger	it	551
anger	in	537
anger	like	475
anger	me	442
anger	im	426
anger	was	420
anger	but	417
anger	so	412
anger	with	403
anger	for	385
love	i	2,641
love	feel	1,180
love	and	1,119
love	to	1,049
love	the	969
love	a	731
love	of	609
love	that	575
love	my	508
love	feeling	471
love	like	393
love	in	380
love	for	344
love	it	336
love	is	284
love	you	266
love	me	265
love	im	251
love	have	244
love	about	229
surprise	i	1,168
surprise	feel	462
surprise	and	439
surprise	the	417
surprise	to	345
surprise	a	307
surprise	feeling	256
surprise	that	252
surprise	of	226
surprise	my	187
surprise	it	171
surprise	in	164
surprise	was	144
surprise	so	118
surprise	like	117
surprise	with	115
surprise	im	113
surprise	but	112
surprise	is	106
surprise	me	104
fear	i	3,782
fear	feel	1,479
fear	to	1,358
fear	and	1,339
fear	the	1,224
fear	a	970
fear	feeling	924
fear	of	763
fear	that	643
fear	my	642
fear	in	474
fear	it	455
fear	about	409
fear	was	397
fear	im	383
fear	but	360
fear	so	354
fear	am	335
fear	me	323
fear	like	311
joy	i	1,0727
joy	feel	4,942
joy	and	4,143
joy	to	4,072
joy	the	3,767
joy	a	2,689
joy	that	2,367
joy	of	2,024
joy	feeling	1,949
joy	my	1,724
joy	in	1,559
joy	it	1,308
joy	like	1,256
joy	is	1,075
joy	have	1,069
joy	im	1,007
joy	for	1,004
joy	so	1,000
joy	am	938
joy	me	937

Table 8 and Table 9 show the full PMI rankings and scores.

**Table 8:** Top-20 bigrams per emotion (real data)

Emotion	Bigram	Frequency
sadness	i feel	2,568
sadness	i am	637
sadness	feel like	618
sadness	i was	516
sadness	and i	425
sadness	i have	410
sadness	that i	410
sadness	feel so	360
sadness	to feel	277
sadness	im feeling	261
sadness	like i	260
sadness	when i	258
sadness	but i	255
sadness	in the	234
sadness	of the	233
sadness	i can	232
sadness	a little	222
sadness	to be	203
sadness	because i	200
sadness	i just	199
anger	i feel	1,147
anger	i am	317
anger	feel like	305
anger	i was	270
anger	that i	194
anger	i have	186
anger	and i	186
anger	im feeling	145
anger	but i	139
anger	to feel	134
anger	when i	129
anger	like i	121
anger	to be	115
anger	feel so	112
anger	of the	109
anger	in the	108
anger	was feeling	105
anger	i can	99
anger	a little	98
anger	i just	95
love	i feel	727
love	feel like	235
love	i am	185
love	that i	135
love	to be	117
love	i have	113
love	and i	110
love	to feel	105
love	i was	99
love	feel that	90
love	in the	76
love	but i	76
love	like i	73
love	i can	71
love	im feeling	70
love	of the	64
love	passionate about	62
love	of my	60
love	feel so	57
love	feel the	55
surprise	i feel	274
surprise	i was	83
surprise	i am	82
surprise	feel like	60
surprise	that i	52
surprise	i have	49
surprise	and i	47
surprise	a little	45
surprise	feel a	37
surprise	at the	35
surprise	of the	34
surprise	in the	34
surprise	but i	33
surprise	feeling a	33
surprise	a bit	32
surprise	to be	32
surprise	when i	30
surprise	im feeling	29
surprise	i m	28
surprise	like i	27
fear	i feel	876
fear	i am	312
fear	i was	265
fear	that i	160
fear	a little	159
fear	i have	156
fear	feel like	155
fear	and i	150
fear	im feeling	132
fear	feeling a	129
fear	of the	124
fear	a bit	122
fear	when i	117
fear	feel a	110
fear	am feeling	108
fear	feel so	104
fear	but i	103
fear	in the	100
fear	i can	100
fear	was feeling	99
joy	i feel	3,143
joy	i am	874
joy	feel like	765
joy	i have	527
joy	that i	501
joy	i was	439
joy	and i	439
joy	to be	413
joy	im feeling	365
joy	of the	347
joy	in the	318
joy	to feel	304
joy	feel that	299
joy	feel so	270
joy	like i	264
joy	i can	260
joy	when i	259
joy	am feeling	250
joy	but i	244
joy	in my	205

**Table 9:** PMI (word, emotion) with frequency of ≥5 PMI: pointwise mutual information

Emotion	Word	PMI Score
sadness	listless	1.8337
sadness	unimportant	1.8337
sadness	victimized	1.8337
sadness	unsuccessful	1.8337
sadness	esteem	1.8337
sadness	disheartened	1.8337
sadness	perverse	1.8337
sadness	sorrowful	1.8337
sadness	submissive	1.8337
sadness	gloomy	1.8337
sadness	moronic	1.8337
sadness	inadequate	1.8337
sadness	remorseful	1.8337
sadness	disliked	1.8337
sadness	idiotic	1.8337
sadness	doomed	1.8337
sadness	disillusioned	1.8337
sadness	defective	1.8337
sadness	pained	1.8337
sadness	ties	1.8337
anger	angered	2.8746
anger	furious	2.8746
anger	heartless	2.8746
anger	envious	2.8746
anger	spiteful	2.8746
anger	irate	2.8746
anger	vicious	2.8746
anger	insincere	2.8746
anger	aggravated	2.8746
anger	vile	2.8746
anger	infuriated	2.8746
anger	savage	2.8746
anger	dissatisfied	2.8535
anger	rebellious	2.8476
anger	greedy	2.8376
anger	resentful	2.8356
anger	hostile	2.834
anger	grouchy	2.8328
anger	bitchy	2.8322
anger	dangerous	2.8309
love	slutty	3.5083
love	treasured	3.5083
love	amorous	3.5083
love	affectionate	3.5083
love	sympathetic	3.4884
love	loyal	3.4827
love	compassionate	3.461
love	horny	3.4602
love	tender	3.4422
love	fond	3.4208
love	longing	3.4195
love	delicate	3.4152
love	nostalgic	3.4136
love	gentle	3.3909
love	naughty	3.389
love	supportive	3.3827
love	devoted	3.3614
love	romantic	3.2933
love	caring	3.2859
love	kisses	3.2452
surprise	enthralled	4.7597
surprise	impressed	4.7008
surprise	stunned	4.7008
surprise	dazed	4.6941
surprise	curious	4.6629
surprise	amazed	4.6429
surprise	shocked	4.5775
surprise	surprised	4.4209
surprise	funny	4.308
surprise	pleasantly	3.8853
surprise	silent	3.7597
surprise	experiment	3.7597
surprise	ludicrous	3.7597
surprise	sentence	3.5373
surprise	overwhelmed	3.4504
surprise	weird	3.4421
surprise	awe	3.4378
surprise	combined	3.4378
surprise	demands	3.4378
surprise	agent	3.4378
fear	distraught	3.1025
fear	neurotic	3.1025
fear	startled	3.1025
fear	bashful	3.1025
fear	petrified	3.1025
fear	timid	3.1025
fear	wimpy	3.1025
fear	apprehensive	3.0832
fear	shaken	3.0824
fear	pressured	3.0815
fear	shaky	3.0787
fear	skeptical	3.0715
fear	distressed	3.0686
fear	suspicious	3.066
fear	reluctant	3.0588
fear	intimidated	3.0581
fear	hesitant	3.0374
fear	frantic	3.0369
fear	alarmed	3.0285
fear	terrified	3.0237
joy	reassured	1.5484
joy	superior	1.5484
joy	elegant	1.5484
joy	splendid	1.5484
joy	festive	1.5484
joy	graceful	1.5484
joy	smug	1.5484
joy	energetic	1.5484
joy	bouncy	1.5484
joy	artistic	1.5484
joy	invigorated	1.5484
joy	contented	1.5484
joy	glamorous	1.5484
joy	keen	1.5484
joy	suggestions	1.5484
joy	clever	1.5484
joy	intelligent	1.5484
joy	glamourous	1.5484
joy	tranquil	1.5484
joy	terrific	1.5484

Table 10 shows the full per-class precision, recall, and F1.

**Table 10:** Machine learning baseline model performance (real data) SVM: support vector machine

Model	Accuracy	Macro Precision	Macro Recall	Macro-F1
SVM	0.891	0.8482	0.829	0.8378
Logistic regression	0.8705	0.8624	0.7696	0.8054

## References

[1] Acheampong FA, Nunoo-Mensah H, Chen W (2021). Transformer models for text-based emotion detection: a review of BERT-based approaches. Artif Intell Rev.

[2] Khurana D, Koli A, Khatter K, Singh S (2023). Natural language processing: state of the art, current trends and challenges. Multimed Tools Appl.

[3] Deng J, Ren F (2021). A survey of textual emotion recognition and its challenges. IEEE Trans Affect Comput.

[4] Min B, Ross H, Sulem E (2023). Recent advances in natural language processing via large pre-trained language models: a survey. ACM Comput Surv.

[5] Plaza-del-Arco FM, Curry AA, Curry AC, Hovy D (2024). Emotion analysis in NLP: trends, gaps and roadmap for future directions. Proceedings of the 2024 Joint International Conference on Computational Linguistics, Language Resources and Evaluation (LREC-COLING 2024).

[6] Gao K, Xu H, Gao C, Hao H, Deng J, Sun X (2018). Attention-based BiLSTM network with lexical feature for emotion classification. IEEE.

[7] Gholipour Shahraki A, Zaïane OR (2017). Lexical and learning-based emotion mining from text. Proc CICLing.

[8] Bandhakavi A, Wiratunga N, Massie S, Padmanabhan D (2017). Lexicon generation for emotion detection from text. IEEE Intell Syst.

[9] Alslaity A, Orji R (2024). Machine learning techniques for emotion detection and sentiment analysis: current state, challenges, and future directions. Behav Inf Technol.

[10] Younis EM, Mohsen S, Houssein EH, Ibrahim OA (2024). Machine learning for human emotion recognition: a comprehensive review. Neural Comput Appl.

[11] Saif WQ, Alshammari MK, Mohammed BA, Sallam AA (2024). Enhancing emotion detection in textual data: a comparative analysis of machine learning models and feature extraction techniques. Eng Technol Appl Sci Res.

[12] Ghafoor Y, Jinping S, Calderon FH, Huang YH, Chen KT, Chen YS (2023). TERMS: textual emotion recognition in multidimensional space. Appl Intell (Dordr).

[13] Nandwani P, Verma R (2021). A review on sentiment analysis and emotion detection from text. Soc Netw Anal Min.

[14] Amadi CO, Odii J, Okpalla C, La OC (2023). Emotion detection using a bidirectional long-short term memory (BiLSTM) neural network. Int J Cur Pharm Rev Res.

[15] Rezapour M (2024). Emotion detection with transformers: a comparative study. arXiv:2403.15454.

[16] Cai Y, Li X, Li J (2023). Emotion recognition using different sensors, emotion models, methods and datasets: a comprehensive review. Sensors (Basel).

[17] Adoma AN, Henry NM, Chen W (2020). Comparative analyses of BERT, RoBERTa, DistilBERT, and XLNet for text-based emotion recognition. IEEE.

[18] Jain S, Wallace BC (2019). Attention is not explanation. Proceedings of the 2019 Conference of the North American Chapter of the Association for Computational Linguistics: Human Language Technologies, Volume 1 (Long and Short Papers).

[19] Wagner J, Andre E, Lingenfelser F, Kim J (2011). Exploring fusion methods for multimodal emotion recognition with missing data. IEEE Trans Affect Comput.

[20] Kusal S, Patil S, Kotecha K, Aluvalu R, Varadarajan V (2021). AI based emotion detection for textual big data: techniques and contribution. Big Data Cogn Comput.

[21] Sun C, Qiu X, Xu Y, Huang X (2019). How to fine-tune BERT for text classification?. Chinese Computational Linguistics.

[22] Boutouta A, Lakhfif S, Senator S, Mediani M (2025). A transformer-based hybrid model for implicit emotion recognition in Arabic text. Eng Technol Appl Sci Res.

[23] Ruiz GB, Herrera RD (2025). Textual emotion detection with complementary BERT transformers in a condorcet’s jury theorem assembly. Knowl-Based Syst.

[24] Jim JR, Talukder MA, Malakar P, Kabir MM, Nur K, Mridha MF (2024). Recent advancements and challenges of NLP-based sentiment analysis: a state-of-the-art review. Nat Lang Process J.

[25] Siriwardhana S, Kaluarachchi T, Billinghurst M, Nanayakkara S (2020). Multimodal emotion recognition with transformer-based self supervised feature fusion. IEEE Access.

[26] Demszky D, Movshovitz-Attias D, Ko J (2020). GoEmotions: a dataset of fine-grained emotions. Proc ACL.

